# Intraperitoneal injection of thalidomide attenuates bone cancer pain and decreases spinal tumor necrosis factor-α expression in a mouse model

**DOI:** 10.1186/1744-8069-6-64

**Published:** 2010-10-05

**Authors:** Xiaoping Gu, Yaguo Zheng, Bingxu Ren, Rui Zhang, Fengmei Mei, Juan Zhang, Zhengliang Ma

**Affiliations:** 1 Department of Anesthesiology, Affiliated Drum-Tower Hospital of Medical College of Nanjing University, Nanjing 210008, Jiangsu province, China

## Abstract

**Background:**

Tumor necrosis factor α (TNF-α) may have a pivotal role in the genesis of mechanical allodynia and thermal hyperalgesia during inflammatory and neuropathic pain. Thalidomide has been shown to selectively inhibit TNF-α production. Previous studies have suggested that thalidomide exerts anti-nociceptive effects in various pain models, but its effects on bone cancer pain have not previously been studied. Therefore, in the present study, we investigated the effect of thalidomide on bone cancer-induced hyperalgesia and up-regulated expression of spinal TNF-α in a mouse model.

**Results:**

Osteosarcoma NCTC 2472 cells were implanted into the intramedullary space of the right femurs of C3H/HeJ mice to induce ongoing bone cancer related pain behaviors. At day 5, 7, 10 and 14 after operation, the expression of TNF-α in the spinal cord was higher in tumor-bearing mice compared to the sham mice. Intraperitoneal injection of thalidomide (50 mg/kg), started at day 1 after surgery and once daily thereafter until day 7, attenuated bone cancer-evoked mechanical allodynia and thermal hyperalgesia as well as the up-regulation of TNF-α in the spinal cord.

**Conclusions:**

These results suggest that thalidomide can efficiently alleviate bone cancer pain and it may be a useful alternative or adjunct therapy for bone cancer pain. Our data also suggest a role of spinal TNF-α in the development of bone cancer pain.

## Background

Primary bone cancers and cancers that metastasize to bone often cause severe pain in humans [1,2]. This pain is dull and constant, increases with time and is exacerbated by use of involved portions of the skeleton. Tumor growth within the bone is accompanied by release of inflammatory and tumorigenic components as well as infiltration and compression of peripheral nerves, and these factors contribute to the development of both ongoing and movement-evoked pain [3,4]. Due to its complex nature and multifactor origin, bone cancer pain is difficult to manage and is relatively resistant to morphine [5,6]. Hence, novel and more efficacious therapies are urgently needed to deal with this condition.

Thalidomide is a relatively selective TNF-α synthesis inhibitor which has been shown to inhibit TNF-α production in vivo [7] and in vitro [8]. The ability of thalidomide to inhibit TNF-α production, has been associated with its clinical benefits in the treatment of many immune inflammatory diseases. Previous studies have demonstrated that thalidomide exerts anti-nociceptive effects in rat models of neuropathic [9,10] and inflammatory pain [11,12], and that the effects are due to the inhibition of TNF-α production by resident inflammatory cells. Moreover, thalidomide can also ameliorate pain-related behaviors produced by taxol chemotherapy [13]. However, the effects of thalidomide on bone cancer pain have not been previously investigated. The aim of the present study was to evaluate the efficacy of thalidomide on bone cancer pain in an established mouse model, produced by injecting NCTC 2472 cells into the intramedullary space of mouse femur [14].

It has been demonstrated that TNF-α is up-regulated in the spinal cord during inflammatory [15] and neuropathic pain [16]. Intrathecal administration of TNF-α induces mechanical and thermal hyperalgesia and enhances responses to C-fibre stimulation, wind-up phenomenon and post-discharge of wide-dynamic range neurons in the spinal cord dorsal horn of anaesthetized rats [17,18]. Overexpression of TNF-α in spinal cord astrocytes exacerbates mechanical allodynia induced by spinal nerve transection [19]. Furthermore, intrathecal administration of the soluble form of p75 Tumor necrosis factor receptor (TNFR) (etanercept) reduces pain-related behaviors in animal models of inflammation [20] and neuropathic pain [21]. These data support a role for spinal cord TNF-α in the development of inflammatory and neuropathic pain. However, whether spinal TNF-α is involved in cancer-induced pain has not been investigated. Therefore, in this study we also detected the expression of spinal TNF-α in bone cancer pain and the effects of thalidomide on bone cancer-induced TNF-α expression, hypothesizing that thalidomide significantly inhibits bone cancer-induced hyperalgesia and concomitantly suppresses TNF-α expression in the spinal cord.

## Results

### Pain behaviors over time

**Figure 1:**
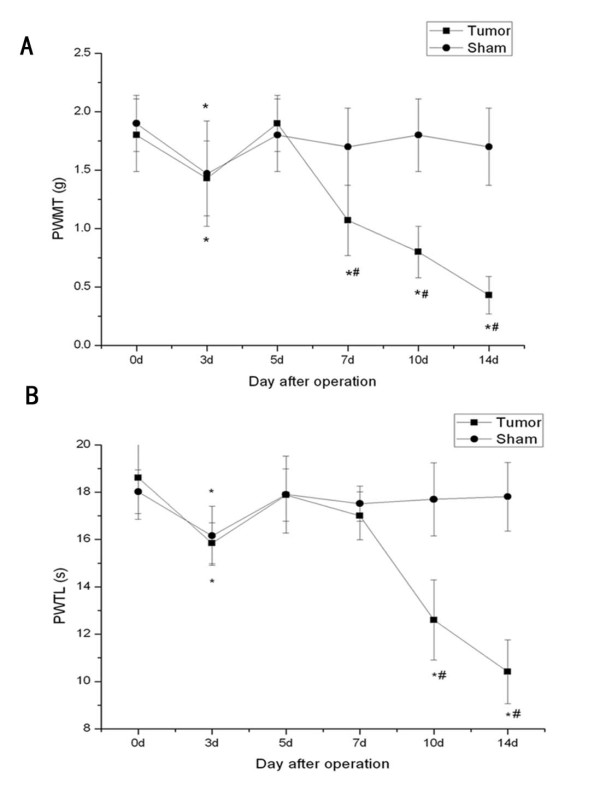
**Changes of pain behaviors of the right hind limb over time in tumor-bearing mice and sham mice**. (A) PWMT to von Frey filaments of tumor-bearing mice decreased as time went on after day 7. (B) PWTL of tumor-bearing mice decresaed gradually after day 10. 0, 3, 5, 7,10 and 14 d indicate days after NCTC2472 cells innoculation to the femur. Data are expressed as means ± SD. ^#^*P *< 0.05 vs sham mice, *P < 0.05 vs day 0.

The ipsilateral hind limb to the operation of both tumor and sham mice displayed a significant decrease of paw withdrawal mechanical threshold (PWMT) to von Frey filaments stimulation at day 3 and the PWMT recovered to the level of day 0 at day 5 (Fig. 1A). At day 7, Tumor-bearing mice then showed a decrease of PWMT of the ipsilateral hind limb which gradually decreased over time until day 14, when the value was 0.43 ± 0.16 g.

The ipsilateral hind limb of both tumor and sham mice displayed the decrease of paw withdrawal thermal latency (PWTL) to radiant heat stimulation at day 3, and recovered to the level before operation at day 5 (Fig. 1B). This transient decrease of PWMT and PWTL at day 3 might attribute to the arthrotomy. PWTL of the tumor-bearing mice at day 10 showed a significant decrease and gradually decreased along with the development of bone cancer pain. At day 14, the value was 10.41 ± 1.35 s in tumor-bearing mice. In addition, no significant differences in PWTL and PWMT were observed in sham mice between days 5, 7, 10, 14 and day 0.

### Effects of intraperitoneal administration of thalidomide on pain behaviors induced by bone cancer pain

**Figure 2:**
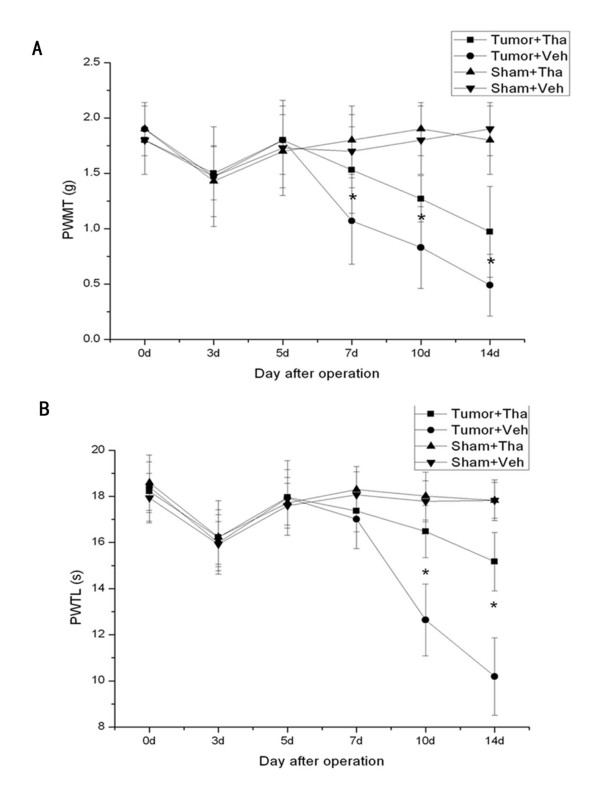
**The effect of intraperitoneal administration of thalidomide on pain-related behaviors produced by bone cancer pain**. PWMT to von Frey filament stimulation (A) and PWTL to thermal stimulation stimulation (B) were ameliorated after treatment with thalidomide. Thalidomide (50 mg/kg) was applied on 1 d after operation and then once daily thereafter until day 7. Thalidomide alone did not affect the behavior response in sham mice (A, B). All data points represent mean ± SD (n = 6 per group). * P < 0.05 vs tumor + vehicle group.

The paw withdrawal thresholds between tumor receiving thalidomide group and tumor receiving vehicle group were not significantly different until day 7 after operation (Fig. 2A). Compared with tumor receiving vehicle group (1.07 ± 0.39) g, PWMT of tumor receiving thalidomide group increased to 1.53 ± 0.39 g at day 7. Although drug treatment was discontinued at day 7, this anti-allodynia effect was maintained throughout day 14.

PWTL of tumor-bearing mice was ameliorated by intraperitoneal administration of thalidomide (Fig. 2B). At day 10 after operation, compared with tumor receiving vehicle group (12.64 ± 1.56) s, PWTL of tumor receiving thalidomide group extended to 16.48 ± 1.13 s. And the analgesic effect of thalidomide continued to exist at day 14. Thalidomide itself had no effect on thermal and mechanical nociceptive threshold in sham mice.

### Measurement of TNF-α expression in the spinal cord of tumor-bearing mice

**Figure 3:**
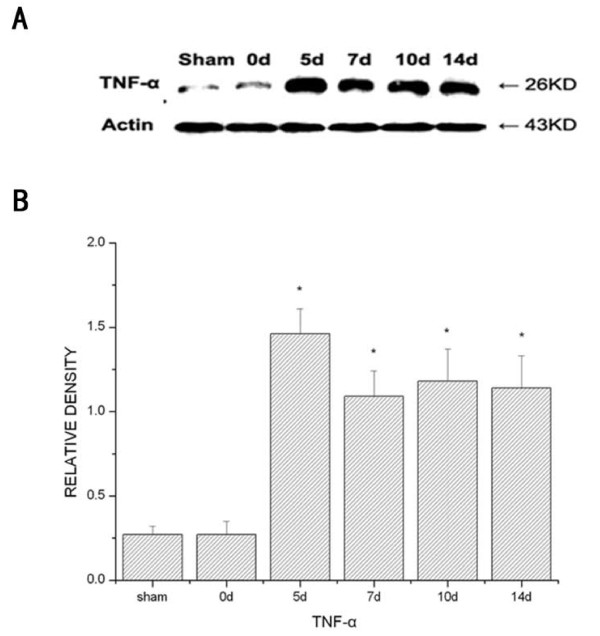
**Time course of spinal TNF-α changes after tumor cells innoculation to the bone**. Tumor induced up-regulation of the TNF-α expression within the spinal cord. (A) Bands of Western blot of the TNF-α expression (26 KDa). β-actin is a loading control. (B) Statistical analysis of relative density of Western blot between tumor and sham mice. * P < 0.05 vs sham mice.

The expression of TNF-α was significantly up-regulated in cancer mice compared with that of sham control, as revealed by Western blot. (Fig. 3A, B). Compared with sham mice (0.27 ± 0.05) and preoperative levels (0.27 ± 0.08), spinal TNF-α levels were increased at day 5 (1.46 ± 0.15) after operation and this increase persisted at day 7 (1.09 ± 0.15), day 10 (1.18 ± 0.09) and day 14 (1.14 ± 0.19). No significant differences in TNF-α levels were observed between sham mice and preoperative levels.

### Effects of intraperitoneal administration of thalidomide on spinal TNF-α expression

**Figure 4:**
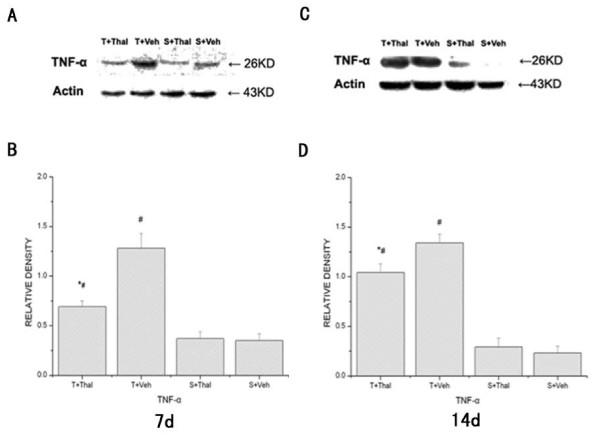
**Spinal TNF-α changes after intraperitoneal administration of thalidomide**. Thalidomide decreased the expression of TNF-α in the spinal cord at day 7 (A, B) and 14 (C, D) after operation. (A, C) Bands of Western blot of the TNF-α expression (26 KDa). β-actin is a loading control. (B, D) Statistical analysis of relative density of Western blot between four groups. * P < 0.05 vs tumor + vehicle group, ^#^P < 0.05 vs sham + vehicle group.

Intraperitoneal administration of thalidomide decreased the expression of TNF-α in the spinal cord at day 7 (Fig. 4A, B) and 14 (Fig. 4C, D) after operation. At day 7, spinal TNF-α levels of tumor receiving thalidomide group (0.69 ± 0.06) was significantly decreased compared with that of tumor receiving vehicle group (1.28 ± 0.15). Although drug treatment was discontinued on day 7, compared with tumor receiving vehicle group, tumor receiving thalidomide group still showed a decrease in the expression of TNF-α at day 14 after operation. There were no differences in TNF-α expression between sham receiving thalidomide mice and sham receiving vehicle mice.

## Discussion

Pain is a common and debilitating symptom of cancer. Tumor growth is accompanied by infiltration and compression of peripheral nerves as well as release of inflammatory and tumorigenic components, and these factors contribute to the generation of both ongoing and movement-evoked pain. TNF-α is a potent pro-inflammatory cytokine which can be produced by tumor cells and recruited inflammatory cells at the tumor site.

Recent evidence suggests that TNF-α may affect bone cancer-related hyperalgesia. In the calcaneus cancer pain model, Wacnik et al [22] found that TNF-α was released at the tumor site and systemic pre-implantation as well as local post-implantation injection of the soluble receptor antagonist TNFR:Fc could partially block ongoing tumor-associated mechanical hyperalgesia. Constantin et al [23] reported that TNF-α induced cancer-related heat hyperalgesia was linked to upregulation of transient receptor potential vanilloid 1 (TRPV1), and systemic treatment with etanercept could prevent tumor-induced nociceptor sensitization. Moreover, in TNFR knock-out mice, a significant reduction in tactile allodynia is observed after tumor cell inoculation [24]. In addition, there is in vitro evidence that TNF-α produced by cancer cells may be responsible for stimulation of osteoclast activity and the resulting bone resorption [25]. These data suggest that local production of TNF-α may contribute to tumor-induced nociception and raise the possibility that TNF-α antagonists or receptor blockers may be useful in treating cancer pain. Accordingly, first case reports show analgesic effects of TNF antagonists in patients with treatment-refractory pain caused by bone metastases [26]. Similarly, in our study, we found that intraperitoneal injection of TNF-α synthesis inhibitor thalidomide was effective in reducing tumor-induced mechanical allodynia and thermal hyperalgesia. Our results provide further support for the use of TNF-α antagonism as a means of reducing cancer pain, particularly bone cancer pain.

Many previous studies have shown the antinociceptive effect of thalidomide in several models of peripheral inflammation [11,12,27] and nerve injury [9,10,16]. The antinociceptive effect correlated with the inhibition of TNF-α production. Interestingly, in these studies, only pre-emptive treatment with thalidomide could attenuate mechanical allodynia and thermal hyperalgesia. Moreover, other drugs, including pentoxifylline and etanercept, which inhibit production of TNF-α or of its receptors, show anti-inflammatory and analgesic properties only when given preemptively [28,29]. These data provide evidence that TNF-α may only participate in the initiation of local inflammation and neuropathic pain [30]. Our previous studies have shown that the establishment of the mouse femur cancer pain model takes approximately two weeks [31,32]. Therefore, in the present study, thalidomide was treated in the initial seven days after operation, which is similar with the preemptive treatment. We also found that thalidomide had no significant effect on PWMT and PWTL of sham mice, excluding non-specific effects of thalidomide on pain perception.

In our study, we found thalidomide treatment attenuated the behavioral effects of bone cancer pain but did not completely reverse them. We may not have selected the optimal dose of thalidomide or, more likely, apart from TNF-α, there may be involvement of additional factors in bone cancer pain. For instance, other cytokines like interleukin-1 may also contribute to the development of pain in this animal model [33].

Spinal glial activation has been observed in several bone cancer pain models [14,34,35]. Activated glial cells are associated with the increased release of proinflammatory cytokines, which may be implicated in the hyperalgesia induced by injecting cancer cells into the bone [36]. The present study further demonstrated that spinal TNF-α was significantly up-regulated in tumor-bearing mice and intraperitoneal injection of thalidomide decreased spinal TNF-α expression as well as bone cancer-induced mechanical allodynia and thermal hyperalgesia. These data are consistent with previous reports that spinal TNF-α is up-regulated during inflammatory [15] and neuropathic pain [16]. Intrathecal administration of etanercept reduces pain-related behaviors in animal models of inflammation [20] and neuropathic pain [21]. In the present study, we provide additional evidence that bone cancer-induced TNF-α up-regulation may be responsible for the development of mechanical allodynia and thermal hyperalgesia. However, as thalidomide is only a relatively selective TNF-α synthesis inhibitor that can also inhibit interleukins (IL) 1β, 6, 12, and granulocyte macrophage-colony stimulating factor (GM-CSF) [37]. Moreover, the drug was administered systematically in our study. Hence, further studies with intrathecal TNF-α receptor antagonists are necessary to demonstrate the potential role of TNF-α in the development of bone cancer pain.

Consistent with our study, previous studies have suggested that systematic treatment with thalidomide suppresses spinal TNF-α expression during neuropathic pain [16] and spinal cord ischemia/reperfusion injury [38]. We suppose that there are two possible mechanisms that may contribute to the decrease of spinal TNF-α after treatment with thalidomide. First, as thalidomide has good central nervous system penetration, it may act directly on the glial cells in the spinal cord and decrease the synthesis of TNF-α. Second, thalidomide inhibits TNF-α release at the tumor site and reduces peripheral nociceptive inputs to reach the spinal cord, which may inhibit glial activation and subsequent spinal release of proinflammatory cytokines.

## Conclusions

In summary, the present study demonstrated that bone cancer induced mechanical allodynia and thermal hyperalgesia as well as the up-regulation of TNF-α in the spinal cord. We found that TNF-α synthesis inhibitor thalidomide decreased the bone cancer related pain behaviors as well as the up-regulation of TNF-α in the spinal cord. Our study suggests that thalidomide may be an effective novel option for the treatment of bone cancer pain. We also provide evidence that spinal TNF-α may participate in the development of bone cancer pain.

## Methods

### Animals

Adult (4-6 weeks old) male C3H/HeJ mice weighing 20-25 g were used for this study. Animals were obtained from the Model Animal Research Center of Nanjing University, housed on a 12-hour light/dark schedule, and allowed ad libitum access to food and water. All experiments were approved by the Animal Care and Use Committee at the college and were in accordance with the guidelines for the use of laboratory animals [39]. All efforts were made to minimize animal suffering and to reduce the number of animals used.

### Cell culture and implantation

Osteosarcoma NCTC 2472 cells (American Type Culture Collection, ATCC, 2087787) were incubated and subcultured in NCTC 135 medium (Sigma-Aldrich, St. Louis, USA) with 10% horse serum (Gibco, Grand Island, USA) at 37°C in an atmosphere of 5% CO2 and 95% air (Thermo Forma, Ohio, USA), and passaged twice a week according to ATCC recommendations.

The mouse model of bone cancer pain was performed as previously described by Schwei [14]. Briefly, mice were anesthetized with intraperitoneal injection of 50 mg/kg pentobarbital sodium (1% in normal saline), and a superficial incision was made in the skin overlying the right articulatio genu with eye scissors. Gonarthrotomy was performed, exposing the femur condyles. A light depression was made using a dental bur. A 30-gauge needle was used to perforate the cortex, then a 25 μl microsyringe was used to inject a volume of 20 μl α-minimum essential medium (α-MEM) containing no or 10^5 ^NCTC 2472 cells into the intramedullary space of the femur, corresponding to sham or tumor-bearing mice. Afterwards, the injection hole was sealed with dental amalgam, followed by copious irrigation with normal saline. The wound was then closed.

### Drug preparation

Thalidomide (Tocris Bioscience, Missouri, USA) was dissolved in dimethylsulfoxide (DMSO) and then diluted in saline to a final concentration of 10 mg/ml (the concentration of DMSO was 10%, v/v). 10% DMSO was also used for vehicle treatment. In the thalidomide treated groups, thalidomide was injected (50 mg/kg) intraperitoneally (i.p.) at day 1 after operation and then daily thereafter until the 7th day. The control group received corresponding volume of vehicle injection.

### Experimental protocol

78 mice were used in the study. The study included four independent experiments and different animals were used in each experiment.

### Experiment 1: pain behaviors over time

We examined the time course of changes in behavior after tumor inoculated to the bone. Withdrawal threshold and latency to mechanical and thermal stimulation respectively were examined during a 2-week period: day 0 before operation and on days 3, 5, 7, 10 and 14 after operation in both tumor-bearing mice (n = 6) and sham (n = 6) mice.

### Experiment 2: Effects of intraperitoneal administration of thalidomide on pain behaviors induced by bone cancer pain

To examine the effects of thalidomide on bone cancer-induced pain behaviors, thalidomide was intraperitoneally given at day 1 after operation and then once daily for 7 days. Four groups of mice (n = 6/group) were used, which included: (1) tumor + vehicle; (2) tumor + thalidomide (50 mg/kg); (3) sham + vehicle; (4) sham + thalidomide (50 mg/kg). Thermal hyperalgesia and mechanical allodynia were tested on day 0 (baseline) and postoperative days 3, 5, 7, 10, and 14.

### Experiment 3: Measurement of TNF-α expression in the spinal cord of tumor-bearing mice

To determine whether bone cancer would change the expression of TNF-α in the L_3_-L_5 _spinal cord, we used five groups of mice (n = 3/group) to obtain tissue samples (Western blot) on day 0 and postoperative day 5, 7, 10, or 14 from tumor-bearing mice. One group of sham-operated mice (n = 3) was used as control, and their spinal cord samples were removed on postoperative day 14.

### Experiment 4: Effects of intraperitoneal administration of thalidomide on the expression of TNF-α in the spinal cord

To determine whether thalidomide would change the expression of TNF-α in the L_3_-L_5 _spinal cord, eight groups of mice (n = 3/group) were used, which included: (1) tumor + vehicle (7 d, 14 d); (2) tumor + thalidomide (7 d, 14 d); (3) sham + vehicle (7 d, 14 d); (4) sham + thalidomide (7 d, 14 d). Tissue samples (Western blot) were obtained at day 7 and 14 after operation.

### Mechanical allodynia

Mechanical allodynia was assessed by using von Frey filaments (Stoelting, Wood Dale, IL) as described by Tal and Bennett [40]. Animals were placed in individual plastic boxes (10×10×15 cm) on a metal mesh floor and allowed to acclimatize for 30 min. Mechanical threshold was measured using a set of von Frey filaments (0.16 g, 0.4 g, 0.6 g, 1.0 g, 1.4 g, 2.0 g). The filaments were perpendicular to the plantar surface with sufficient force to cause slight bending against the paw and were held for 6-8 s with a 10-min interval between each stimulation. The positive response was defined as a withdrawal of hind paw upon the stimulus. The paw withdrawal mechanical threshold (PWMT) was determined by sequentially increasing and decreasing the stimulus strength. Each mouse was tested 5 times per stimulus strength. The lowest von Frey filaments which had 3 or more positive responses were regarded as PWMT.

### Thermal hyperalgesia

Thermal hyperalgesia to radiant heat was determined according to a method described by Hargreaves et al [41]. In brief, mice were placed in clear plastic cages on an elevated glass plate and allowed to acclimatize for 30 min before testing. A radiant thermal stimulator (BME410A, Institute of Biological Medicine, Academy of Medical Science, China) was focused onto the plantar surface of the hindpaw through the glass plate. The nociceptive end-points in the radiant heat test were the characteristic lifting or licking of the hindpaw, and the time to the end-point was considered the paw withdrawal thermal latency (PWTL). The cutoff time of 20 s was used to avoid tissue damage. There were five trials per mouse and 5 min intervals between trials. The mean PWTL was obtained from the latter three stimuli.

### Western Blotting

Mice were rapidly (< 1 min) killed through decapitation after being anesthetized with pentobarbital, and the L_3_-L_5 _lumbar spinal cord segments were removed rapidly and stored in liquid nitrogen. Tissue samples were homogenized in lysis buffer. The homogenate was centrifuged at 13,000 rpm for 10 min at 4°C and supernatant was removed. The protein concentration was determined by the BCA Protein Assay Kit, following the manufacturer's instructions. Samples (50 μg) were separated on SDS-PAGE (12%) and subsequently transferred to polyvinylidene difluoride membranes (Millopore Corporation, MA, USA). The filter membranes were blocked with 5% nonfat milk for 1 h at room temperature and incubated with goat anti-TNF-α primary antibody (1:500, Santa Cruz Biotechnology, CA, USA). The membrane was washed with TBST buffer and incubated for 1 h with the secondary antibody conjugated with horseradish peroxidase (1:5000; BioVision, CA, USA) for 1 h at room temperature. Next, the immune complexes were detected using the ECL system (Santa Cruz Biotechnology, CA, USA). β-actin was used as a loading control for total protein. The images of Western blot products were collected and analyzed by Quantity One V4.31 (Bio-Rad, USA).

### Statistical Analysis

All data are expressed as mean ± SD (standard deviation). Repeated measures ANOVA was performed to determine overall differences at each time point in PWMT and PWTL. One-way ANOVA was used to determine differences in the expression of TNF-α among all experiment groups. In both cases, when significant main effects were observed, LSD post hoc tests were performed to determine the source(s) of differences. A P value < 0.05 was considered statistically significant.

## List of abbreviations used

TNF-α: Tumor necrosis factor α; PWMT: paw withdrawal mechanical threshold; PWTL: paw withdrawal thermal latency; TNFR: Tumor necrosis factor receptor; TRPV1: transient receptor potential vanilloid 1; DMSO: dimethylsulfoxide.

## Competing interests

The authors declare that they have no competing interests.

## Authors' contributions

All authors read and approved the manuscript. XPG, YGZ and BXR carried out the administration of drugs, surgical procedure and western blots studies and drafted the manuscript; RZ and FMM were responsible for pain behavioral tests and statistical analysis; JZ participated in the design of the study; ZLM conceived the idea, designed the study and helped to draft the manuscript for this study.
